# Curcumin, but not its degradation products, in combination with silibinin is primarily responsible for the inhibition of colon cancer cell proliferation

**DOI:** 10.17912/micropub.biology.000617

**Published:** 2022-07-28

**Authors:** Alhan Sayyed, Rita Heuertz, Uthayashanker R Ezekiel

**Affiliations:** 1 Nutrition and Dietetics; 2 Saint Louis University, St. Louis, MO; 3 Clinical Health Sciences

## Abstract

Colorectal cancer (CRC) is the third leading cause of cancer death globally and the most-commonly diagnosed cancer in men and women in the United States. We have previously shown that the phytochemicals curcumin, derived from turmeric, and silibinin from milk thistle exhibit synergistically enhanced anticancer activity against colorectal cancer cells. In the present study, the combination of curcumin, a major component of turmeric, and its degraded products trans-ferulic acid, ferulic aldehyde, and vanillin in combination with silibinin were assessed for their action against cancer cell proliferation. Our results indicate that only curcumin plus silibinin has significant antiproliferative effects on colon cancer cells.

**Figure 1. Antiproliferative effect of Curcumin and Curcumin Degradation Products in Combination with Silibinin.**
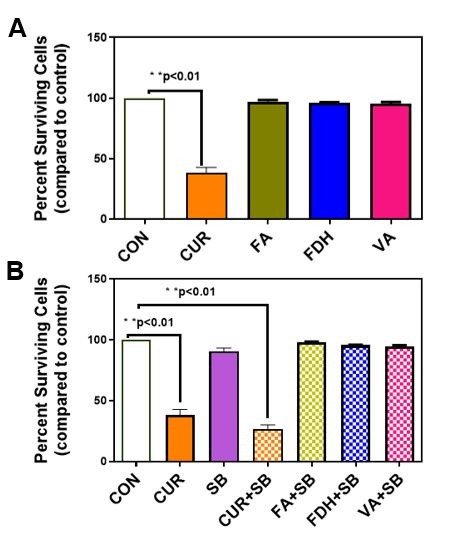
(A) Antiproliferative effect of curcumin (CUR) and curcumin degraded products, trans-ferulic acid (FA), ferulic aldehyde (FDH), and vanillin (VA). DLD-1 cells were treated with 12.5µM of the compounds and incubated for 48hrs. Percent surviving cells were compared to control. Statistically significant reduction in surviving cells was observed only in curcumin-treated cells. **<0.01, n=3. B). (B). Antiproliferative effect of curcumin, curcumin degraded products in combination with silibinin. DLD-1 cells were treated with 12.5µM of curcumin (CUR) or 12.5µM of curcumin degraded products (FA, FDH, VA) plus 12.5µM of silibinin (SB) and incubated for 48hrs. Percent surviving cells were compared to control. Statistically significant reduction in surviving cells was observed only in the curcumin plus silibinin-treated cells. **<0.01, n=3.

## Description

Colorectal cancer (CRC) is a leading cause of death globally and is the third leading cause of cancer-related deaths in the USA (Sung et al. 2021). Alarmingly, a notable increase in colorectal cancer rates has been observed in the USA in the under 50 age group (Weinberg and Marshall 2019). It is predicted that by 2030, the rate of CRC in the 20-34 year-old age group will increase to 90% and 124% (colon and rectal, respectively) (Bailey et al. 2015), and significantly, a sharp increase in CRC in the 18-35 year-old group is already being observed (Bailey et al. 2015, Loomans-Kropp and Umar 2019, Weinberg and Marshall 2019). Current treatment for CRC is based on tumor removal followed by radiotherapy and/or chemotherapy (Kaiser et al. 2014). Unfortunately, both treatments cause adverse side effects and increase chemoresistance and the rate of metastasis (Kawamoto et al. 2012, Liu et al. 2021, Vilalta et al. 2018). They can also be detrimental to a patient’s overall health due to high levels of toxicity. Several studies have shown that a diet high in fruits, vegetables, and spices is strongly associated with a reduced risk of developing CRC (Vernia et al. 2021). Although individual phytochemicals may not exhibit significant anticancer activity against CRC cells, combinations of different phytochemicals could produce additive or synergistic anticancer effects against CRC. In comparison to drug therapy, treatments involving combinations of phytochemicals could be effective at lower, non-toxic doses, thus making them practical treatment options for CRC prevention and suppression. Curcumin is the primary curcuminoid in the spice turmeric, which comes from the dried rhizome of *Curcuma longa* (Pricci et al. 2020). Silibinin is the active constituent isolated from seeds of milk thistle, *Silybum marianum* . Silibinin (also known as silybin) is the major component of silymarin and is traditionally used for liver diseases (Hogan et al. 2007). We previously showed (Montgomery et al. 2016) that a combination of curcumin and silymarin exhibited synergistic anticancer activity.

Curcumin has been shown to have multiple biological activities, such as anti-inflammation, anti-bacterial, anticancer, anti-arthritic, with several possible therapeutic applications (Gupta et al. 2012, Hatcher et al. 2008, Shen et al. 2016). The use of curcumin in therapy is hindered by its poor availability (Shehzad et al. 2010a, Shehzad et al. 2010b). In spite of this poor bioavailability, there are several *in vitro* and *in vivo* studies showing remarkable biological effects mediated by curcumin (Shen et al. 2016). One possibility is that curcumin degraded products may manifest these effects. In a physiological aqueous solution, curcumin can be degraded to trans-ferulic acid ferulic aldehyde, and vanillin (Gordon and Schneider 2012, Shen et al. 2016, Tsuda 2018, Typek et al. 2019). Our previous result observed with curcumin and silymarin could be attributable to some of the degraded products eliciting the combination effect. In order to ascertain if the degradation products of curcumin are responsible for the anticancer effects on CRC cells, we tested similar concentrations of curcumin (IC _50_ ) and the degraded product in combination with silibinin and measured cell death, with the controls being without any drug.

Curcumin IC _50_ value for DLD-1 cells was 12.5uM (Montgomery et al. 2016). We treated cells with individual compounds at 12.5uM concentration or in combination with silibinin for 48hrs, and the number of viable cells was compared to the vehicle control using the crystal violet method (Basile et al. 2013, Duessel et al. 2008, Montgomery et al. 2016). In this study, we used three degradation products of curcumin, trans-ferulic acid, ferulic aldehyde, and vanillin . When DLD-1 cells were treated with the individual compounds, only curcumin exhibited a significant inhibition of cell growth (Figure 1A). When we compared percent growth compared to control, curcumin exhibited significantly higher inhibition of cell growth (38±4, Figure 1A). None of the other curcumin degradation products showed any inhibition of cell growth (Figure 1A).

Next, we treated DLD-1 cells with 12.5uM silibinin or 12.5uM silibinin with 12.5uM curcumin or curcumin degradation products, trans-ferulic acid, ferulic aldehyde, and vanillin. When we compared the percent growth, only curcumin plus silibinin exhibited significant inhibition of cell growth (26±3, Figure 1B). The other three curcumin degraded products, trans-ferulic acid, ferulic aldehyde, and vanillin in combination with silibinin did not show inhibition of cell growth when compared to control (figure 1B).

In the present study we showed that the curcumin degradation products in combination with silibinin did not inhibit cell growth compared to the curcumin plus silibinin combination. Our result supports our previous study that curcumin and silibinin in combination inhibit cell growth significantly (Montgomery et al. 2016). Among the curcumin degraded products, either singly or in combination with silibinin, none of them showed significant inhibition of cell growth compared to control.

Epidemiological studies show that populations consuming larger amounts of spices and vegetables have a low incidence of CRC (Aggarwal and Shishodia 2006, Baena and Salinas 2015, Kotecha et al. 2016, Li et al. 2015, Rizeq et al. 2020). For example, in Asian Indians, the overall rates of colorectal, prostate, and lung cancers in both males and females are the lowest among populations studied, and this low incidence is attributed to the phytochemical curcumin present in the spice turmeric (Pricci et al. 2020, Sinha et al. 2003). However, when these phytochemicals have been studied *in vitro* and translated into mice and human studies, the pharmacologically effective dose is several-fold higher than that indicated by epidemiological studies (Pricci et al. 2020). For example, in clinical trials, curcumin has shown promising anticancer effects and is well-tolerated at a dose of 12 g/day (Carroll et al. 2011, Epstein et al. 2010, Lao et al. 2006). But the average dietary intake of turmeric in the Asian population is about 2- 2.5 g/day which corresponds to 60-100 mg/day of curcumin (Chainani-Wu 2003, Sharifi-Rad et al. 2020). In addition, the low bioavailability of curcumin has led to questions about how epidemiological studies and several research publications can show medicinal effects in animal and human studies against several diseases, such as cancer, Alzheimer’s, and chronic diseases, such as arthritis (Baliga et al. 2012, Carroll et al. 2011, Daily et al. 2016, Dhillon et al. 2008, Gupta et al. 2013, Padmanaban and Nagaraj 2017, Shen et al. 2016). Curcumin can be degraded under physiological conditions, and several studies suggested it could be the degraded products of curcumin that function as bioagents (Shen et al. 2016). Despite the low bioavailability of curcumin and the relatively low daily dietary intake (Shen et al. 2016, Teiten et al. 2010, Tsuda 2018), the beneficial effect of curcumin observed could be due to other phytochemicals present in the diet and act synergistically in cancer prevention. We have published data showing that a combination of curcumin and silymarin (CS), elicited synergistically enhanced anticancer activity *in vitro* (Montgomery et al. 2016). In the present study, we showed it is curcumin but not the curcumin degradation products that elicit the combination anticancer effects with silibinin. Our future studies will be focused on the mechanism by which curcumin plus silibinin causes the anticancer effects using *in vitro* and *in vivo* systems.

## Methods

Cell proliferation assay: DLD-1 cells were propagated (37°C, 5% CO _2_ ) until confluent. Cells were treated with trypsin (0.25%) to detach them from culture plates, followed by the addition of enriched DMEM to neutralize trypsin. Cells (5000 cells/100 μl) were added to each well of 96-well microplates and incubated overnight to allow cells to attach. Cells were treated with 12.5uM of single compounds, curcumin, silibinin, trans-ferulic acid, ferulic aldehyde, and vanillin either alone or in combination with 12.5uM of silibinin. Cell proliferation was assessed using a previously described crystal violet method (Basile et al. 2013, Duessel et al. 2008, Montgomery et al. 2016).

All treatments were performed in quadruplicate, and each experiment was performed at least three times independently. In addition to treatment wells, control wells were assessed, which included cell blanks (medium only), and vehicle controls (untreated cells plus media plus DMSO at the highest concentration assessed). At 48 h, cells were fixed by addition of glutaraldehyde (20 μL, 11% glutaraldehyde, 15 min rotation, 300 rpm), washed with water, dried, and stained (0.1% crystal violet). Crystal violet that was not cell-bound was removed by washing (3 water washes), and then the plates were dried. Cell-bound crystal violet was solubilized (10% acetic acid), and absorbance was determined in a microplate reader (562 nm).

## Reagents

Human colon cancer cell line DLD-1 was obtained from the American Type Culture Collection (Manassas, VA). The cells were cultured in Dulbecco’s modified Eagle’s medium (DMEM) supplemented with 10% fetal bovine serum, penicillin/streptomycin, glutamine, sodium pyruvate, and HEPES buffer. DMEM and culture medium supplements were purchased from Hyclone (Logan, UT). Curcumin, silibinin, trans-ferulic acid, ferulic aldehyde, and vanillin (Sigma-Aldrich, Inc, St. Louis, MO) were prepared and stored in DMSO as 100 mM stock solutions.
